# Systematic Review of the Respiratory Syncytial Virus (RSV) Prevalence, Genotype Distribution, and Seasonality in Children from the Middle East and North Africa (MENA) Region

**DOI:** 10.3390/microorganisms8050713

**Published:** 2020-05-11

**Authors:** Hadi M. Yassine, Muhammad U. Sohail, Nadin Younes, Gheyath K. Nasrallah

**Affiliations:** 1 Biomedical Research Center, QU Health, Qatar University, Doha P.O. Box 2713, Qatar; Hyassine@qu.edu.qa (H.M.Y.); msohail@qu.edu.qa (M.U.S.); ny1204022@qu.edu.qa (N.Y.); 2 Department of Biomedical Sciences, College of Health Sciences, QU Health, Qatar University, Doha P.O. Box 2713, Qatar

**Keywords:** RSV, MENA, prevalence, genotype, children age, season, host gender

## Abstract

Respiratory syncytial virus (RSV) is one of the most common viruses to infect children worldwide and is the leading cause of lower respiratory tract illness (LRI) in infants. This study aimed to conduct a systematic review by collecting and reviewing all the published knowledge about the epidemiology of RSV in the Middle East and North Africa (MENA) region. Therefore, we systematically searched four databases; Embase, Medline, Scopus, and Cochrane databases from 2001 to 2019 to collect all the information related to the RSV prevalence, genotype distribution, and seasonality in children in MENA region. Our search strategy identified 598 studies, of which 83 met our inclusion criteria, which cover the past 19 years (2000–2019). Odds ratio (OR) and confidence interval (CI) were calculated to measure the association between RSV prevalence, gender, and age distribution. An overall prevalence of 24.4% (*n* = 17,106/69,981) of respiratory infections was recorded for RSV. The highest RSV prevalence was reported in Jordan (64%, during 2006–2007) and Israel (56%, 2005–2006). RSV A subgroup was more prevalent (62.9%; OR = 2.9, 95%CI = 2.64–3.13) than RSV B. RSV was most prevalent in children who were less than 12 months old (68.6%; OR = 4.7, 95%CI = 2.6–8.6) and was higher in males (59.6%; OR = 2.17, 95%CI = 1.2–3.8) than in female infants. Finally, the highest prevalence was recorded during winter seasons in all countries, except for Pakistan. RSV prevalence in the MENA region is comparable with the global one (24.4% vs. 22%). This first comprehensive report about RSV prevalence in the MENA region and our data should be important to guide vaccine introduction decisions and future evaluation.

## 1. Introduction

Respiratory syncytial virus (RSV) is a common pathogen that causes acute lower respiratory infections (ALRI). The virus is the leading cause of bronchiolitis and pneumonia, particularly among children younger than one year of age. The WHO estimates that the annual burden of RSV-related ALRI is 33 million globally, with about 3 million hospitalizations and 59,600 in-hospital deaths [1]. About half of these RSV hospitalizations and in-hospital deaths were recorded in infants aged younger than 6 months [2]. More than 93% of all RSV related ALRI and 99% of RSV related deaths occur in developing countries [2]. It is suggested that poor hygiene and lack of access to basic medical care results in high infection and complication rates.

RSV infections show strong seasonal distribution with the highest incidence rate during winter and with the least or no outbreaks during summer. In addition, correlations between virus infection and atmospheric temperature, relative humidity, and rainfall were reported [3]. The virus spreads by aerosol or through the self-inoculation of the nose and eyes [4]. In hospital wards, more than 40% of children may get the infection through direct contact with contaminated fomites and large droplets [5]. Although the infection may present mild-common-cold symptoms, the consequences may be devastating, particularly in children with underlying conditions (premature birth, chronic lung disease, congenital heart disease, or Down’s syndrome) [6,7]. These infants have relatively less developed immune systems, and their innate and acquired immune responses against RSV infections are inadequate [8]. Therefore, it leads to severe morbidity and a substantial increase in health care costs. Some studies report that the disease severity is also subgroup-specific and that the infection caused by the subgroup A (RSV A) is more lethal than for the subgroup B (RSV B) [9,10].

Currently, palivizumab is the drug of choice for treatment and prophylaxis to prevent RSV infection in high-risk children [11]. For several decades, efforts were made to develop and commercialize vaccines that can cover a wide range of RSV strains and patient age groups. Schickli et al. [12] comprehensively described the challenges facing RSV vaccine development since the 1960s, when the first RSV vaccine trial resulted in adverse effects and enhanced disease illness [13]. WHO’s Strategic Advisory Group of Experts on Immunization (SAGE) recognizes a lack of age–genotype-stratified RSV burden records from Africa and Asia, reflecting a page in an evidence-based recommendation to introduce and evaluate potential RSV vaccine candidates [2,14]. Regional and global WHO estimates on RSV incidences predict wide differences in intra-country and inter-regional epidemiological numbers.

The Middle East and North Africa (MENA) region consists of 21 countries that are spread on two continents, Asia and Africa. The term MENA typically includes the area from Morocco in northwest Africa to Iran in southwest Asia and down to Sudan in Africa. The MENA region includes Algeria, Bahrain, Djibouti, Egypt, Iran, Iraq, Israel, Jordan, Kuwait, Lebanon, Libya, Malta, Morocco, Oman, Qatar, Saudi Arabia, Syria, Tunisia, United Arab Emirates, Palestine, and Yemen [15].

Although individual studies estimate a high incidence of RSV in the MENA region, unified data on genotype, seasonality, and age-strata are scarce [16,17,18]. Two comprehensive reports on global and regional RSV prevalence did not include information from the MENA region due to the scarcity of published data [2,19]. Therefore, in the present study, we prepared a systematic review of all published research articles from the MENA region to evaluate RSV prevalence, seasonality, genotypes, and patients’ age and gender data during the period between 2001 to 2019. Our data demonstrate a high prevalence/incidence of RSV in the MENA region. Overall, RSV prevalence is higher in male children under 12 months of age. Only a few studies reported that RSV genotypic prevalence with RSV A subgroup is more dominant than RSV B. Presented data in this study will be beneficial towards the adoption and evaluation of future RSV vaccines.

## 2. Methods 

### 2.1. Search Strategy and Selection Criteria

A systematic literature review of journals published online was conducted. All original articles that reported RSV prevalence and genotyping between 2001 and 2019 in the MENA region countries were collected. Four databases were searched (Embas, Scopus, PubMed MEDLINE, and Cochrane Library). We used a combination of controlled keywords; “respiratory syncytial virus”, “RSV”, “prevalence”, “incidence”, “epidemiology”, “Middle East”, and “individual country name from the MENA region”. The eligible articles were screened for both the titles and abstracts. A further search of the bibliographic lists from relevant articles was made to explore eligible articles. The studies included in this systematic review have been selected based on the following criteria: (i) articles published in peer-reviewed journals, (ii) patients residing in the MENA region, (iii) articles containing data on RSV along with data on other respiratory viral infections. Review articles, case studies, clinical trials, Haj pilgrimage studies, and adult age strata studies were excluded from our analysis.

### 2.2. Data Collection and Data Adjustment

A comprehensive data collection sheet was designed to extract data from the selected articles. A quantitative summary of individual study parameters was prepared using Microsoft Excel. The following information was extracted: article title, publication year, sample size, sampling year, country, seasonality, age, gender of subjects, type of outbreak, diagnostic laboratory procedure, and the RSV genotype. Recorded data were reviewed and confirmed by all the authors. For any unclear data, the other authors were consulted before any data imputations were applied. Not all studies stratified RSV prevalence by gender, seasonality, and uniform age groups. Furthermore, in many cases, the description of seasonality and age groupings were far different. Many studies compared RSV prevalence at different age intervals. However, we observed that most of the studies compared RSV incidence between two age groups: below 12 months (<12 M) and above 12 months of age (>12 M). Similarly, many studies described RSV seasonality according to the month of sampling, whereas a few also compared between Winter, Spring, Autumn, and Summer season. To address the uniformity and to gain statistical inference, we distributed RSV samples into two age groups: <12 M and >12 M. We also described seasonality according to the month of samples’ collection. If age grouping was different from that described above, we added all age strata below 12 months and all age strata above 12 months to make the <12 M and the >12 M groups, respectively. For the seasonality, we only chose those studies which reported monthly prevalence and discarded rest for the seasonality analysis.

From the selected studies, prevalence data were extracted and arranged according to country and year of sample collections. All the data were reported as percentages. A mean of percentage prevalence was taken if more than one prevalence study was reported from the same country for the same year. Similarly, seasonality, gender, age, and RSV genotypes were recorded and presented as mean percentages. Data were compared by Fisher’s exact test, and *p*-values were calculated for chi-square of association. Odds ratios (OR) at 95% confidence intervals (95%CI) were calculated using OpenEpi 3.01 (open source program, Atlanta, USA) [20] and VassarStats (Poughkeepsie, USA) [21] online epidemiologic statistics tools.

## 3. Results

### 3.1. Literature Search and Selection Process

**Figure 1:**
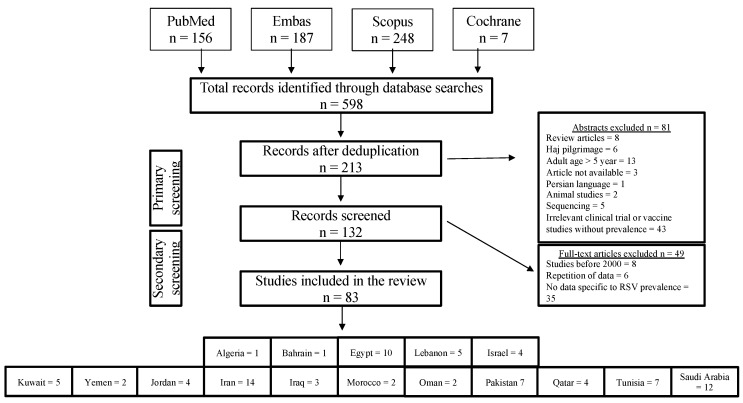
Flowchart of literature search and studies selection. n: number.

The literature review process and selection of relevant studies are described in a flowchart in Figure 1. A total of 598 research records, from 16 MENA region countries, were initially identified by electronic literature searches. 387 research records were excluded after research database duplication. In the primary screening process, 132 research records were further excluded based on title, abstract, and keywords evaluation, and only 83 publications were retained for full-text review. Based on our inclusion/exclusion criteria, only 83 research articles were retained for data retrieval and analysis from 16 countries. All of these articles are listed in Table A1 in Appendix A. Considering the small number of initial electronic literature search records (*n* = 598) originating from the region, we kept our selection criteria relatively loose and included all those studies that reported the prevalence of RSV in children from 2001 to 2019.

### 3.2. RSV Prevalence and Population Demography

**Figure 2:**
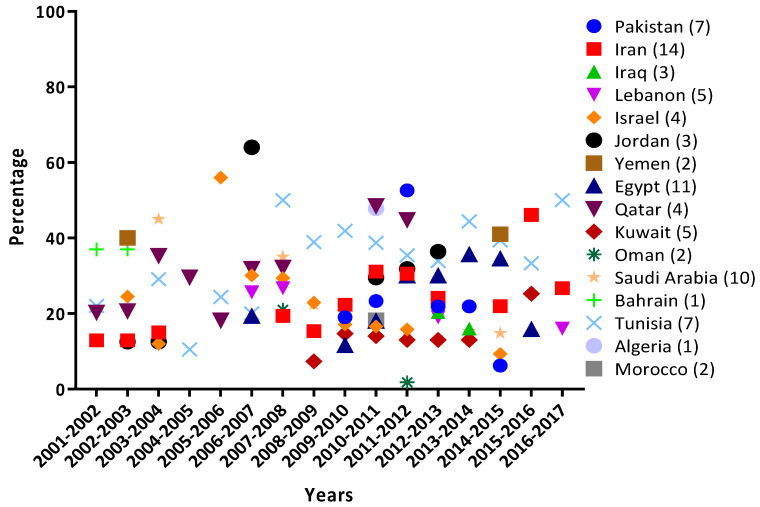
Annual rate of prevalence of Respiratory Syncytial Virus infection in the MENA region detected from 2001–2019.

RSV diagnosis was mostly confirmed using PCR-based molecular techniques (88% of the studies), 5% used enzyme-linked immunosorbent assay (ELISA) 5%, and 7% used immunofluorescence. Nearly half of the reviewed studies (44/83) reported a prevalence of common viral pathogens (including RSV) associated with respiratory illnesses. In total, 69,981 patients were reported in 83 studies, of which 17,106 (24.4%) patients were positive for RSV only, and 34,059 (48.7%) subjects were positive for at least one viral pathogen (including RSV). In addition, 1336 (1.9%) were positive for coinfections. The year-wise prevalence of RSV in different MENA countries is presented in Figure 2. The highest annual prevalence of RSV was reported in Jordan (64.0% during 2006–2007), followed by Israel (56.0% during 2005–2006), Pakistan (52.6% during 2011–2012), Tunisia (50.0% during 2007–2008 and 2016–2017), Qatar (48.5% during 2010–2011), Algeria (47.8% during 2010–2011), Egypt (46.6% during 2013–2014), and Iran (46.1% during 2015–2016). The lowest annual prevalence of RSV (1.8%; 95%CI = 0.91–3.80) was reported from Oman during 2011–2012.

**Figure 3:**
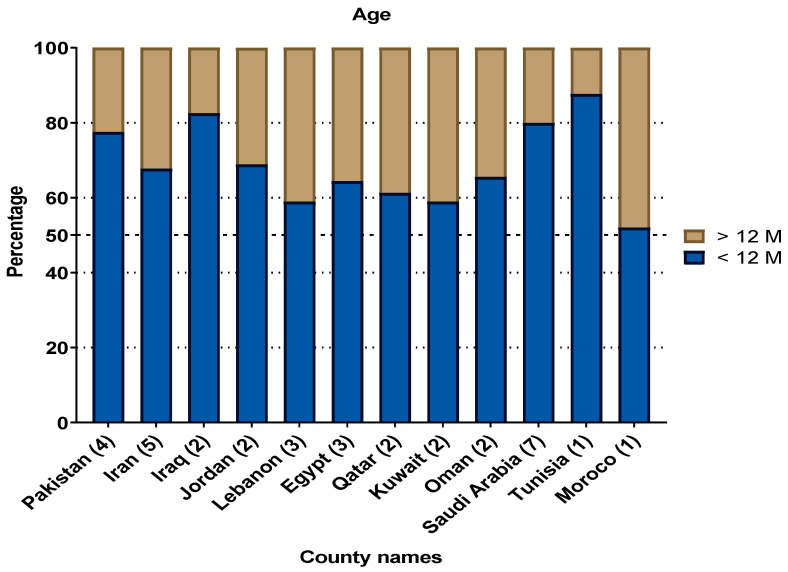
Age distribution of Respiratory Syncytial Virus (RSV) infections in the Middle East and North Africa (MENA) region: under 12 months of age (<12 M) and above 12 months of age (>12 M).

**Figure 4:**
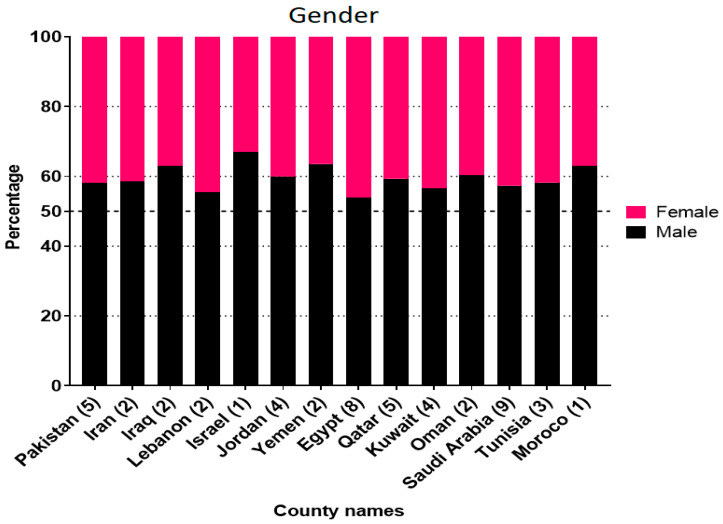
Distribution of Respiratory Syncytial Virus in male and female children.

In total, 53 studies reported the age-wise distribution of the RSV infection. However, 21 studies were excluded from the analysis as they describe only mean or median age of the infection, or otherwise described the overall age grouping for all viral infections associated with respiratory illnesses. The remaining 32 studies that reported the age-wise distribution of RSV prevalence were included in the analysis as presented in Figure 3. Children under 12 months of age (68.6%) were more likely (OR = 4.77, 95%CI = 2.627–8.672; *p* < 0.001) to be infected with RSV than those above than 12 months of age (31.04%). In these 32 studies, 6384 RSV cases were age-stratified, out of which 2176 belonged to Saudi Arabia. Among the studied countries, the highest prevalence of RSV in infants <12 M of age was reported from Tunisia (87.6%), Iraq (82.5%), and Saudi Arabia (79.9%). However, no statistical difference was observed between the age-wise reported RSV infection rates of the studied countries (*p* = 0.058). Finally, overall, RSV prevalence was higher in the male infants (59.6%; OR = 2.17, 95%, CI = 1.23–3.82) than in the females (40.4%) in almost all countries of the MENA (Figure 4).

### 3.3. RSV Monthly Prevalence in the MENA Region

**Figure 5:**
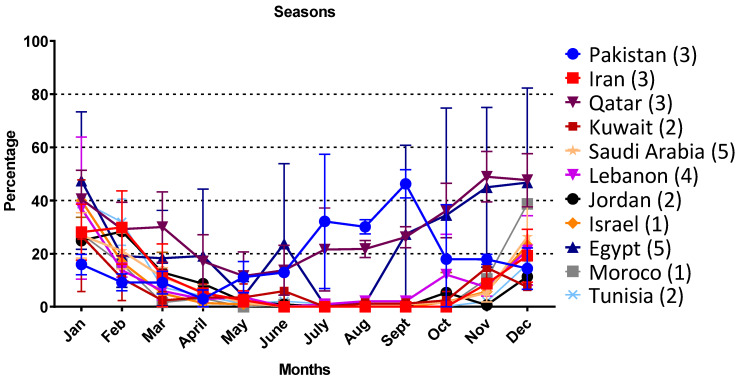
Seasonal distribution of Respiratory Syncytial Virus positive cases.

Thirty studies reported on the monthly prevalence rate of the virus, as presented in Figure 5. The virus prevalence rate was higher (*p* < 0.001) during winter months (November, December, January, and February). Except for Pakistan, RSV prevalence rate was relatively high during monsoon months (July, August, and September).

### 3.4. RSV Subgroups and Strains Circulating in the MENA Region

**Figure 6:**
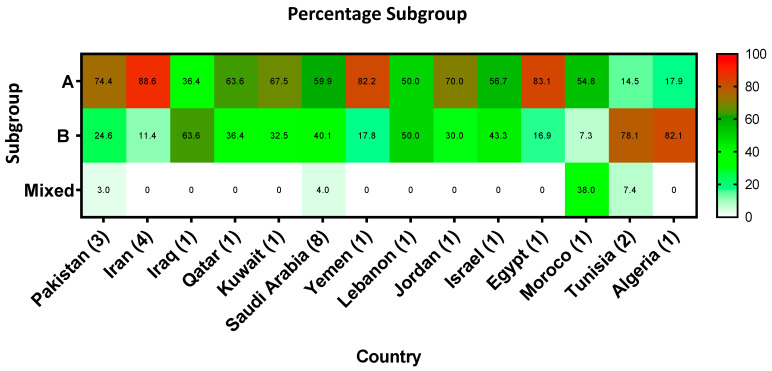
RSV subgroups (RSV A & RSV B) distribution in MENA region between 2001–2019.

**Figure 7:**
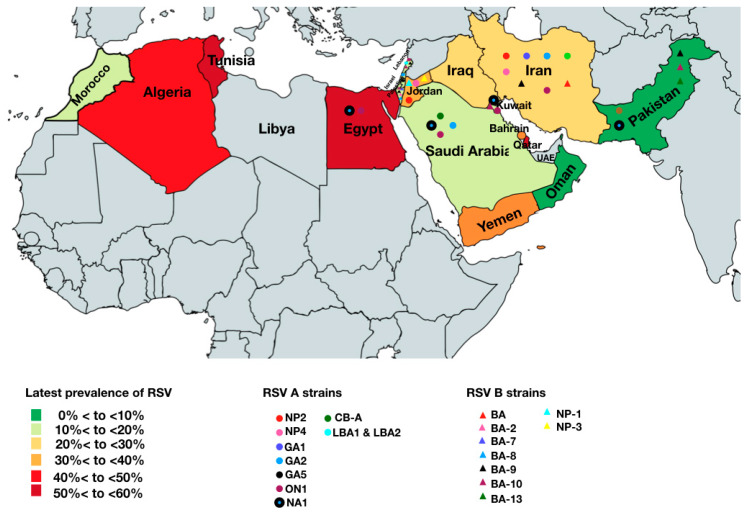
RSV subgroups and strains circulating in the MENA region.

Twenty-seven studies described subgroups and genotypes of 5205 RSV samples in total. Generally, RSV A subgroup (62.9%) was more dominant (OR = 2.87, 95%CI = 2.62–3.13) in the MENA region than the RSV B (37.1%). As shown in Figure 6, RSV A was predominant in Pakistan, Iran, Israel/Palestine, Jordan, Yemen, Egypt, Saudi Arabia, Qatar, Kuwait, and Morocco. Interestingly, RSV B subgroup infections were more dominant (*p* < 0.001) than RSV A infections in Iraq (63.6%), Tunisia (74%), and Algeria (82.1%) (Figure 6). The circulation of RSV A strains (NP1, NP2, GA1, GA2, GA5, ON1, NA1, CB-A, LBA1, and LBA2) and RSV B strains (BA, BA-2, BA-7, BA-8, BA-9, BA-10, and BA-13) in the MENA region fluctuated from 2001 to 2019 (Figure 7 and Table A1). For instance, In Iran and Jordan, NP4 and NP2 were predominant in 2003-2004. Later, GA1 and GA2 was predominant during 2007–2009 and reappeared during 2010–2013 in Iran, Israel/Palestine, Saudi Arabia, and Pakistan. GA5 was only detected in 2008 in Iran and Israel/Palestine [22]. From 2014 to 2015, a striking shift in RSV A circulation patterns from GA2 to ON-1 was reported in Iran, Saudi Arabia, Egypt, Lebanon, and Kuwait as shown in Table A1. Regarding RSV B, the most common genotype was BA, as shown in Table A1. From 2010 to 2017, BA-9 and BA-10 were dominating in Lebanon, Pakistan, Israel/Palestine, and Saudi Arabia. BA-13 was only reported in Pakistan from 2009 to 2013. In addition, NP1 and NP3 were only reported in Jordan in 2003–2004. Moreover, BA-2, -7, and -8 were only reported in Israel/Palestine (Figure 7, Table A1). Interestingly, in 2016, novel strains of RSV B; LBA1 and LBA2, appeared in Lebanon [23].

## 4. Discussion

### 4.1. The prevalence of RSV in the MENA Region

RSV is the most prevalent virus associated with respiratory tract infections in young children and elderly adults. Particularly, the chances of acquiring the infection and developing severe illness are higher in immunocompromised subjects [18]. Currently, palivizumab is the only licensed antiviral drug available for disease prevention and treatment. Premature infants and young children with underlying medical complications are medicated with palivizumab at regular intervals to prevent the disease [24]. However, the drug has relatively low efficacy (34%–51%) and a high investment of resources in the selected population [25]. Currently, more than 60 RSV vaccine candidates are in development, out of which 16 are in the human testing phase [26]. So far, achieving immunogenicity against all RSV strains and balancing between vaccine immunogenicity and safety have proven difficult [12]. Since disease severity was determined by the virus genotype, hostage, health, and immune status, it is strongly desired to initiate global efforts to optimize the disease pathogenesis and clinical features in different populations and environment settings [27].

Because ALRI is one of the leading causes of morbidity and hospitalization in the MENA region, and because the region has the highest population growth rate [28], it is of utmost importance to review and summarize RSV prevalence and demography in this region [29,30]. In this systematic review, eighty-three RSV prevalence studies were analyzed to summarize country-wise prevalence, genotype distribution, seasonal circulation patterns, and other demographic characteristics of the virus. In total, these studies reported about 24.4% annual prevalence of RSV in the MENA region during the study period between 2001–2019. This estimate is slightly higher than the global incidence (22%) of RSV-related ALRI episodes in young children [19]. The highest prevalence of RSV for a single year was reported from Jordan (2006–2007), Israel/Palestine, Pakistan, and Tunisia. On the other hand, few countries reported low prevalence rates around the same period, including Oman (1.8% during 2011–2012) and Kuwait (4% during 2008–2009).

The MENA region countries have wide variations in environment, geography, demographics, and the World Bank GDP index. These environmental and demographic factors were frequently reported in the literature for promoting RSV infections [17]. Shi et al. [18] reported a high incidence rate of RSV and ALRI episodes in low-income countries with poor access to primary health services and hospitalization. The reason for country-wise variation in the prevalence of RSV, as observed to be between 1.8% in Oman and 64% in Jordan [31,32], is not clear but suggests that clinical diagnosis, sampling procedures, method of detection, and access to healthcare services probably played large roles [33]. For instance, the American National RSV Surveillance Data [34] showed that access to a laboratory for RSV screening is the major contributor in RSV reporting during different seasons.

### 4.2. Seasonal Distribution of RSV in the MENA Region

In all the reviewed studies from the MENA region, RSV displayed strong seasonal distribution with high prevalence during the winter season [35]. Cold temperature and high precipitation rate promote virus prevalence, as was observed in our study (Figure 5). Regardless of the variation in climate and demographic characteristics, RSV exhibited higher prevalence during winter seasons in all countries except in Pakistan, where it is relatively higher in monsoon season during July, August, and September months (Figure 5). A similar trend of RSV prevalence is reported from the eastern Indian state, Odisha, where RSV infection shows seasonal variation, with peaks during the rainy season followed by winter season [36]. Similarly, data analysis of the European Influenza Surveillance Network shows a specific seasonal periodicity of RSV infections in Europe, where a major RSV outbreak in winter is followed by a minor outbreak in the spring season [37].

### 4.3. RSV Subgroup and Strains Circulating in the MENA Region

Two subgroups of RSV exist, RSV A and RSV B, based on their reactions with monoclonal antibodies [38]. The RSV viral genome encodes 11 proteins, including G- and F- proteins as the major surface proteins [38,39]. Neutralizing antibodies are secreted against both proteins [40]. However, based on the genetic variability of the G protein, the RSV A is subdivided into 15 strains (GA1–7, SAA1, NA1–4 CB-A, and ON1–2) [41], while the RSV B group is subdivided into 24 strains (SAB1–4, BA1–12, GB1–4, GB5/CB1, CBB, and URU1–2) [42,43]. Thus, the infectivity, immunological resistance, and viral genetic drift (spontaneous mutation) may be important in the patterns of seasonal circulation and genetic evolution of RSV genotypes [44].

Few studies reported on RSV subgroup and genotype distribution. The high prevalence rate was observed for RSV A subgroup in most of the countries, except in Tunisia (74%) and Algeria (82.1%), where RSV B subgroup is more prevalent (Figure 6). The predominance of RSV A has also been described in several other regions, but in a cyclic pattern, where RSV B peaks for a short period and then suddenly declines [45,46,47]. Such a cyclic pattern was missing in our report, principally due to a lack of continuous RSV surveillance programs in the region. In our report, only one study from Israel/Palestine presented RSV A and RSV B subgroups cocirculation for seven consecutive years from 2005 to 2012 [48]. This study reported that RSV A virus was predominant during four epidemic seasons (2005, 2007, 2009, and 2010), while RSV B virus was dominant during the subsequent 2006, 2008, and 2011 epidemic seasons. A common factor that explains this periodicity is not known; however, natural infection from one episode may provide limited protective immunity owing to the evolution of the surface protein G and alternate dominance of antigenic groups A and B [49]. Another reason could be due to the limited variability among RSV B, which might contribute to a more protracted spread of these viruses, leading to the predominance of RSV A over RSV B viruses. Concurrently, it is also an established fact that various RSV subgroups and strains may co-circulate during one season, and the predominant strain may change from year to year [50,51,52,53,54,55,56].

In Iran, 66.6% of the positive samples belonged to RSV A and 33.4% to RSV B. Phylogenetic analysis revealed that RSV A strains fell in two clusters, GA1 and GA2, where all RSV B strains clustered in BA genotype with a 60-nucleotide insertion in the second variable region of the G protein during the season 2009 [57]. However, there was a striking shift in RSVA circulation patterns from GA2 to ON-1 from 2015 to 2016 [58].

In Lebanon, Abou-El-Hassan and his colleagues demonstrated that during the 2016/17 season, two distinct lineages of RSV were co-circulating, ON1 and BA9, with the temporal disappearance of NA2 and BA10 genotypes [23]. The RSV A ON1 genotype has a 72-nucleotide duplication that was identified initially in Canada and spread worldwide thereafter, likely due to the fitness advantage of this strain. Interestingly, they reported two novel genotypes named LBA1 and LBA2 that descended from the ON1 and NA2 genotypes, respectively [23]. LBA1 genotype is characterized by six amino acid substitutions and possesses an additional O-glycosylation site (G284S) compared to the reported ON1 genotype. Meanwhile, LBA2, a descendant of the NA2 genotype, was characterized by two amino acid substitutions and an additional O-glycosylation site (S292) compared to NA2. None of the sequences reported in the database belonged to the LBA1 genotype and its geographic spread is yet to be determined [23].

The ON1 genotype of RSV A was also detected in Egypt, where a high detection rate of RSV A was reported in hospitalized infants with lower respiratory tract infections. Two genotypes were found, ON1 and N1, with a predominance of genotype ON1 train in 2014–2015. However, four amino acid substitutions in the original Canadian viruses, including L274P, L298P, Y304H, and L310P, occurred in the Egyptian ON1 genotypes [59]. These novel mutations are considered noteworthy because the adjacent region (aa 265–273) is a reported antigenic site [53]. Another variant was also reported in 11 out of 15 patients in Egypt, which is the P310L amino acid substitution. This variant has been associated with the abrogation of the reaction of peptides to convalescent-phase human serum [60]. In addition, Abdel-Moneim et al. reported that some of these changes would cause the loss of a site, while others would cause site gains [59]. These sites were found to be epitopes in escape mutants either screened with specific monoclonal antibodies [61,62,63] or in naturally isolated strains [50,60,61]. In Egypt, two unique amino acid substitutions were detected among Egyptian strains: Thr 253 Lys and Phe 265 Leu. The latter was found to be an epitope described in escape-mutant strains [59].

In Israel/Palestine [48], RSV subgroup A was dominant around October/ November of the 2005–2006, 2006–2007, and 2007–2008 winter seasons (*p* < 0.0005). From 2008, no particular dominant genotype was detected; both RSV subgroups A and B were detected in relatively high percentages throughout the winter season (*p* < 0.05). Before 2008, the phylogenetic analysis revealed that most of the RSV A genotypes were either GA5 (40%) or GA2 (60%), with GA2 dominating [48], and all of the RSV B genotypes were clustered in the BA genotype. After 2008 until 2012, the GA2 genotype of RSV A was the most dominant in the country (95.2%). In parallel, until 2008, the BA7/8/9/10 genotype co-circulated in the country, where BA9 comprised about 44% of the RSV B infections, BA7 and BA8 led to approximately 24% of the infections, and BA10 accounted for only 10% of the hospitalized patients [48]. In contrast, from 2008 and on, fewer patients were infected with BA8 and BA10 (6%) genotypes, while BA7 genotype was not detected at all. After that, another strain appeared, BA9 (80%) genotype, which became the dominant genotype. Interestingly, by 2009–2010, RSV infections declined. The delay in RSV infections could be attributed to the emergence of the pandemic infection of the H1N1 influenza virus, as similar delays were observed in other countries [48,64,65,66].

In Kuwait, Madi et al. [67] reported that all RSV A strains collected from the patients were untyped genotypes that did not belong to any of the known strains of RSV A in the GenBank database. Besides, the data showed that these Kuwaiti strains formed different clusters of identical sequences. These data indicate that there is heterogeneity among the Kuwaiti strains, which differ from the known RSV A strains [67]. To investigate whether these strains were new, they conducted whole-genome sequencing [67]. They found out that the RSV A Kuwaiti strains were more closely related to the new RSV A/ON1 genotype [67]. They also reported that the RSV B/BA10 genotype was the predominant strain among Kuwaiti RSV B strains, while the rest of the Kuwaiti RSV B strains formed three clusters of untyped genotypes [67]. RSV B/BA genotype emerged in the late 1990s and then spread globally and became the predominant strain in Kuwait [67,68]. Other studies have also demonstrated the predominance of RSV B/BA genotype in the MENA region, which is in line with our findings [22,23,48,57,69].

RSV is an important viral pathogen among hospitalized children in Saudi Arabia [70]. Most studies investigated RSV prevalence in Saudi Arabia detected RSV infections in 20%–25% of the respiratory samples [69,70,71,72,73], which correlates well with most of the studies conducted in the Middle Eastern countries [27,57,74] and internationally [46,75]. RSV A predominated over RSV B in Saudi Arabia [69,70,71,72,73]. Ahmed et al. reported that the phylogenetic analysis clustered the RSV A positive strains in the NA1 and ON1 genotypes, with 82.6% belonging to the NA1 genotype [70]. The NA1 genotype was also reported in a study from Riyadh by Almajhdi et al. [73]. In addition, Ahmed et al. reported that all the diagnosed RSV B sequences belonged to the BA genotype, with 60 bp duplication in the second hypervariable region of the G protein gene [70].

In Qatar, four G gene sequences representing RSV A strains were reported in 2000 and 2001 and were included in Almajhdi et al.’s study [73]. They reported that all RSV A strains in Qatar appear to cluster within the genotype GA2, where they tend to form a separate branch from the NA-1 and CB-A genotypes.

A study conducted in Jordan showed that RSV is the most common cause of ALRI in young children. As shown in most MENA countries, RSV A was predominant compared to RSV B in Jordan [76]. Similar to Iran [77], NP4 and NP2 were the predominant genotypes of RSV A in the study period 2003–2004 in Jordan [76].

In Tunisia, Fodha et al. [78] observed the monthly distribution of RSV groups in Tunisia from 2000 and 2001. Interestingly, unlike the MENA countries, they reported that RSV B was predominating in the outbreak of 2000–2001 [78,79]. Another study was conducted in Tunisia in 2007, in which they also reported the predominance of RSV B over RSV A [80]. However, 14.8% of strains remained un-typeable [80]. A similar pattern was observed in Algeria in 2010, in which Derar et al. reported a higher prevalence of RSV B (82%) [81]. Nevertheless, in the RSV outbreak of 2013–2015 in Saudi Arabia, a similar pattern was observed Egypt and Iraq [82,83,84].

In Pakistan, all the RSV A strains analyzed clustered with viruses ascribed to the previously reported NA1 [85]. On the other hand, three strains of Pakistani RSV B viruses were seen: BA-9 and BA-10, which have been reported previously from other regions, and a new genotype, assigned as BA-13, which formed a distinct cluster [85].

As seen in Table A1, every few years, the existing predominant genotype was replaced by a new genotype. In the MENA region, we found out that GA2 genotype and its related genotypes (particularly NA-1) are the most geographically distributed. They represent the dominant genotypes identified in most epidemics worldwide from 1990 and 2009: Canada, 98.2% [54]; China, 97% [86]; Croatia, 82.9% [87]; Korea, 96.4% [46]; and Japan, 100% [88]. In addition, GA2 is the sole genotype that can persist in communities for long periods without being replaced by another genotype: 20 years in Sweden [89], 13 years in the USA [90], and 6 years in Argentina [91]. After that, the RSV A genotypes GA2, GA5, and GA7 were replaced by NA1 and NA2, while BA became the predominant RSV B genotype [92]. By early 2013, ON1 genotype had spread so efficiently that they had nearly replaced other RSV A genotypes [93]. Thus, the ON1 and BA genotypes have been circulating worldwide for the last 6 and 17 years, respectively [54,68]. RSV A/ON1 is a novel genotype that was first described by Eshaghi et al. in Ontario, Canada [54], and this genotype later emerged and became the dominant genotype in different countries, including northern Italy, Germany, Thailand, Turkey, and, in the MENA region, Saudi Arabia, Iraq, Egypt, Lebanon, Kuwait, Iran, and Pakistan [58,59,69,82,85,93,94,95,96,97,98]. The genetic variations of ON1 and BA genotype occur due to mutations especially in the duplicated region and changes in the stop codon usage leading to the formation of subgroups among themselves [92,99,100]. Antigenic variations may occur in RSV due to changes in the pattern and frequency of glycosylation [52,101]. The rapid antigenic changes in viruses (duplications, deletions) may assist in immune evasion, thus providing an additional advantage to the virus, resulting in their spreading to different geographical regions [102].

### 4.4. Coinfection RSV in the MENA Region

It has been reported that the unique characteristic of RSV facilitates infection with a second respiratory virus [103,104]. Previous studies using RT-PCR techniques reported viral coinfection rates of 5%–10%, with RSV, human rhinovirus (hRV), parainfluenza virus (PIV), and Human metapneumovirus (hMPV) being the most commonly implicated viruses in cases of mixed infections [105,106,107]. The identification of two or more viruses in a patient may be due to prolonged viral shedding or asymptomatic persistence of viruses [108]. Studies have shown hMPV and RSV coinfection rates of approximately ~5%–14% [109,110]. In Saudi Arabia, viral coinfections were detected in 6.7% of the patients. They reported that coinfection of RSV with hMPV (4/9; 44.4%) and hRV with hCoV (2/9, 22.2%) in viral coinfections was an interesting finding [106]. Unexpectedly, in Kuwait, mixed detection was not identified between RSV and hMPV. However, they found out that coinfections with RSV and rhinovirus were the most commonly detected mixed infections among the patients [67]—a finding consistent with those of previous studies [111,112,113,114]. In addition, a study reported that 17 out of 77 positive RSV samples (22%) had mixed infections of RSV and other respiratory viruses [67]. Another study in Kuwait showed that HCoV-OC43 positive patients were most commonly coinfected with RSV [115]. In Iran, Shatizadeh et al. [116] detected 11 coinfections in 202 patients younger than 6 years, and most of the dual infections observed were in combination with RSV. Thus, they speculated that a possible synergy between RSV and the other viruses might exist, leading to cocirculation in the community [116]. The coinfections of RSV with other viral infections may influence the severity of respiratory disease in patients suffering from RSV infection. For instance, Calvo et al. reported that coinfection with RSV and other respiratory viruses did not result in greater severity of the disease, but did result in mixed clinical features between both viral infections [114]. Furthermore, Goka and his colleagues reported an increased risk of admission to the intensive care unit (ICU) and death as a result of coinfection with RSV and other respiratory viruses [117].

### 4.5. Age Distribution of RSV Infections in the MENA Region

We observed a high prevalence rate of RSV in children younger than 12 m of age (68.6%) and males (59.6%). Similar differences in age and gender susceptibility for RSV infection have previously been reported in other regions [47,118,119]. Although the exact reason is unknown, younger age, premature birth, cold temperature, smoking exposure, underlying medical condition, and male gender are known as risk factors for RSV infection [10]. In our analyzed studies, children were less likely to have reported underlying medical conditions or premature birth; the majority of our RSV positive children visited the hospital with evidence of ALRI symptoms (wheezing, cough, pneumonia, bronchiolitis, and retractions) [74,120,121]. However, our observations are in accordance with the global RSV prevalence trend, where about 45% of RSV ALRI are reported in children aged younger than six months [2].

## 5. Conclusions

The respiratory syncytial virus is the leading cause of acute respiratory tract infections in young children in the MENA region. We observed that the virus prevalence and infection demography in the MENA region coincide with the global RSV trends. However, the paucity of nation-wide surveillance data and comparable statistical inferences suggest a clear need for research investment in the field to gather national and region-wide data regarding the burden of RSV ALRI epidemic and pandemic in the region. This review of literature presents a descriptive situation and an initial evidence base to support improved surveillance and reporting in the MENA countries. Better-designed, nation-wide, unselected case series reporting population demography and RSV subgroup and genotype prevalence may substantially improve these estimates and help advance preventive measures, including vaccine development. Further efforts are suggested for molecular epidemiology of RSV over consecutive seasons to observe antigenic and nucleotide level variability in immune epitope regions of RSV A and RSV B groups viruses.

## Appendix A

**Table A1:** The prevalence of RSV and associated demographics in 16 countries of the MENA region.

Country	Study Period	Age Yrs	Sample Size	RSV Positive	Male	Female	Symptoms	Detection Method	Subgroup A	Genotype	Subgroup B	Genotype	Coinfection	Reference
Iran	2001–2003	<5	202	26	NR	NR	Wheezing, cough, fever	RT-PCR	NR	NR	NR	NR	NR	[122]
	2003–2004	<2	261	39	59%	41%	Cough, dyspnea, sneezing, the runny nose and fever	RT-PCR	38 (97.4%)	NP4, NP2	1 (2.6%)	NR	3 (7.7%)	[77]
	2009	<2	107	24	NR	NR	Wheezing, cough, fever	RT-PCR	16 (66.6%)	GA1, GA2	8 (33.4%)	BA	NR	[57]
	2008–2009	<6	202	34	NR	NR	Wheezing, cough, fever	multiplex RT-PCR	NR	NR	NR	NR	11 (32%)	[116]
	2008–2009	<5	100	9	57%	43%	Bronchiolitis, cough,coryza, fever, chest wall retraction, wheezing, cyanosis	RT-PCR	NR	NR	NR	NR	NR	[123]
	2008–2010	<4	180	40	NR	NR	Cough, difficulty in breathing, tachypnea, retraction, crackles and wheezing on lung auscultation.	NR	NR	NR	NR	NR	NR	[124]
	2007–2013	<2	485	94	59%	41%	ALRTI symptoms	RT-PCR	85 (90.43%)	GA1, GA2, GA5	9 (9.57%)	BA		[22]
	2011–2013	<5	280	84	55.9%	44.1%	Bronchiolitis, wheezing, and cough	RT-PCR	NR	NR	NR	NR	10 (11.9%)	[125]
	2010–2013	<5	158	49	44.8%	55.2%	Fever and respiratory distress	RT-PCR	NR	NR	NR	NR	0	[126]
	2012	<5	232	40	67.5%	32.5%	Tachypnea, chest retraction and wheezing	RT-PCR	NR	NR	NR	NR	NR	[127]
	2014–2015	<17	60	5	NR	NR	Wheezing episodes	RT-PCR	NR	NR	NR	NR		[128]
	2014–2015	<15	156	56	43.5%	56.5%	ARI symptoms	RT-PCR	NR	NR	NR	NR	4 (7.1%)	[129]
	2015–2016	<2	180	55	57.8%	42.2%	Cough, dyspnea, sneezing, the runny nose and fever	RT-PCR	55 (100%)	ON-1	0	none	NR	[58]
	2016–2017	<3	75	20	40%	60%	Fever, wheezing, coughing, hypoxia, dyspnea, and rhinorrhea	RT-PCR	NR	NR	NR	NR	4 (20%)	[130]
Lebanon	2004–2014	<5	319	194	56.2%	43.8%	NR	NR	NR	NR	NR	NR	NR	[131]
	2008	<6	120	32	NR	NR	Rhinorrhoea and dyspnea	RT-PCR	NR	NR	NR	NR	NR	[132]
	2008		39	10	NR	NR	Fever ≥38 °C, cough, rhinorrhea, or sore throat	SYBR Green RT-PCR	NR	NR	NR	NR	NR	[133]
	2013–2014	<16	236	45	NR	NR	Fever, cough, runny nose, shortness of breath	RT-PCR	NR	NR	NR	NR	10 (22.2%)	[134]
	2016/17	<3	519	83	54.2%	45.8%	NR	NR	27 (50%)	ON1, LBA1,LBA2 (novel)	27 (50%)	BA9, BA10	NR	[23]
Israel	2003–2004	<5	613	76	NR	NR	Bronchiolitis	RT-PCR analysis and sequencing	NR	NR	NR	NR	NR	[135]
	2005–2006	<2	465	346	NR	NR	NR	NR	NR	NR	NR	NR	NR	[136]
	2005–2006		557	230	NR	NR	Shortage of breath, fever, cough, rhinorrhea, dyspnea, lack of appetite, vomiting, diarrhea and sore throat	RT-PCR	77 (78.6%)	GA5, GA2	21 (21.6%)	BA 2,7,8,9,10	NR	[48]
	2006–2007		617	186					14 (17.1%)		68 (82.9%)		NR	
	2007–2008		558	164					80 (83.6%)		16 (16.7%)		NR	
	2008–2009		903	207					79 (38.0%)		128 (62%)		NR	
	2009–2010		1403	240					139 (59.9%)		95 (40.1)		NR	
	2010–2011		4151	691					324 (67.1%)		159 (32.9%)		NR	
	2011–2012		2829	448					143 (46.1%)		167 (53.9%)		NR	
	2015–2016	0-70	1910	178	NR	NR	fever, cough and dyspnea	RT-PCR	NR	NR	NR	NR	NR	[137]
Yemen	October 2002 to May 2003	<2	604	266	NR	NR	NR	RT-PCR	171 (82%)	NR	37 (18%)	NR	NR	[138]
	2014–2015	NR	1346	552	NR	NR	ALRTI	NR	NR	NR	NR	NR	NR	[139]
Bahrain	2000–2003		235	88	60%	40%	NR	NR	NR	NR	NR	NR	NR	[140]
Saudi Arabia	2003–2004	<1	282	128	57.1%	42.9%	Cough and tachypnea	Direct fluorescein-labeled monoclonal antibody assay.	NR	NR	NR	NR	NR	[141]
	2005–2010	<17	643	295	49.5%	50.5%	NR	Direct immunofluorescence assays	NR	NR	NR	NR	NR	[142]
	2007–2008	<3	200	70	54.3%	45.7%	NR	Monospecific and duplex RT-PCR	40 (57.1%)	NR	30 (42.9%)	NR	NR	[143]
	2008–2009	<3	174	39	66.7%	33.3%	Bronchitis and pneumonia	RT-PCR	23 (58.6%)	NR	16 (41.4%)	NR	8 (20.5%)	[144]
	2007–2009	<3	175	39	NR	NR	NR	multiplex RT-PC	23 (59%)	GA2, NA-1, CB-A	16 (41%)	NR	NR	[73]
	2011	<1	2154	338	69.2	30.8	NR	IMAGEN immunofluorescence test	NR	NR	NR	NR	7 (21.2%)	[145]
	2012–2013	<5	135	33	69.7	30.3	Rhinitis, pharyngitis, cough, earache, hoarseness of voice, rhonchi, crepitations, or wheezy chest	multiplex RT-PCR	30 (90.9%)	NR	3 (9.1%)	NR	NR	[146]
	2012–2013	<13	2235	514	NR	NR	NR	Seeplex RV15 kit	381 (74.4%)	NR	131 (25.6%)	NR	NR	[147]
	November 2013 and January 2014	All ages	182	12	72%	28%	NR	RT-PCR and multiplex microarray	3 (3.4%)	NR	9 (10.2%)	NR	4 (33.3%)	[120]
	2013–2014	<14	4611	1086	54.8	45.2	ARTI symptoms	Immunofluorescence assays	NR	NR	NR	NR	NR	[121]
	2014	<5	130	34	NR	NR	NR	RT-PCR	27 (77%)	NA1, ON1,	8 (23%)	BA9	NR	[69]
	2014–2015	0 to 14 years	2266	336	NR	NR	NR	Anyplex II RV16 detection kit	124 (37%)	NR	212 (63%)	NR	RSV A: 32 (3.7) RSV B: 75(8.7%)	[83]
Iraq	2012–2013	<15	269	55	NR	NR	Fever of ≥38 °C on admission and with clinical signs and symptoms of an upper and/or lower respiratory tract infection	xTAG Respiratory Virus Panel Fast assay	NR	NR	NR	NR	18 (32.7)	[148]
	2013	1-15	80	30	NR	NR	NR	RT-PCR and fluorescent assay	NR	NR	NR	NR	NR	[149]
	2014–2015	<10	250	22	NR	NR	NR	RT-PCR	8 (36.3%)	NR	14 (63.7%)	NR	NR	[82]
Egypt	2006–2007	<5	427	70	NR	NR	Cough and difficult breathing or tachypnea	Immunofluorescent assay (IFA)	NR	NR	NR	NR	none	[74]
	2006–2007	<5	450	107	57.4	41.2	Cough, difficulty breathing, fever, chest indrawing, and rapid breathing	rt-RT-PCR	NR	NR	NR	NR	22 (25.9%)	[150]
	2009–2013	>65	5768	669	NR	NR	Cough, sore throght, tachypnea, sputum production, chest pain, dyspnea	rRT-PCR	NR	NR	NR	NR	NR	[151]
	2010–2011	2-12	130	28	55.6	44.4	cough, tachypnea, sputum, hemoptysis, chest pain, sore throat, and shortness of breath	PCR	NR	NR	NR	NR	none	[152]
	2011–2014	<1	153	69	NR	NR	NR	RT-PCR	NR	NR	NR	NR	NR	[153]
	2013–2014	<2	127	59	47.5	452.5	ALRTI symptoms, severe bronchiolitis or pneumonia, tachypnea, chest indrawing	PCR	11 (18.6%)	NR	46 (78%)	NR	Coinfection of type A and B in 2 patients (3.4%)	[84]
	2010–2014	All ages	3207	485	47	53	Fever, Cough	RT-PCR	NR	NR	NR	NR	3 cases were positive for Mycoplasma and were coinfected with RSV, one case of Chlamydia was coinfected with RSV	[150]
	2014–2015	<5	223	77	NR	NR	NR	RT-PCR	64 (83.1%)	NA1, ON1,	13 (16.8%)	NR	NR	[59]
	2015–2016	<5	120	12	NR	NR	NR	multiplex PCR	NR	NR	NR	NR	6(50%)	[154]
	2016–2017	<2	55	30	61.9	38.1	Cough, tachypnea, wheezes and crackles on auscultation, and hyperinflation	PCR	NR	NR	NR	NR	9 (30%)	[155]
Qatar	2010–2011	2 weeks- 2 years	369	189	59.8	40.2	Bronchiolitis,ever, rhinitis, tachypnoea, cough, wheezing, crackles	PCR	NR	NR	NR	NR	58 (30.7%)	[156]
	2010–2012	<3	770	304	59.5	40.5	Bronchiolitis	RT-PCR	NR	NR	NR	NR	NR	[157]
	2010–2012	<2	769	352	NR	NR	NR	Real-time PCR	NR	NR	NR	NR	NR	[158]
	2002	<2	241	50	20	21.3	Respiratory distress	RT-PCR	NR	NR	NR	NR	NR	[109]
	2003	<2	680	240	32.8	38.9			NR	NR	NR	NR	NR	
	2004	<2	716	212	30.1	29			NR	NR	NR	NR	NR	
	2005	<2	674	123	17.5	19.3			NR	NR	NR	NR	NR	
	2006	<2	470	150	32.4	31.3			NR	NR	NR	NR	NR	
	2007	<2	340	45	13.5	12.9			NR	NR	NR	NR	NR	
Oman	2007–2008	<5	259	56	66	34	Runny nose, cough, sore throat, Earache, Fever, Wheezing, Tachypnoea, Chest indrawing	Multiplex PCR	NR	NR	NR	NR	NR	[159]
	2011–2012	2 months to 13 yrs	373	7	NR	NR	NR	PCR	NR	NR	NR	NR	2 (5.9%)	[32]
Kuwait	2008–2010	<76	1014	106	NR	NR	Bronchiolitis, Croup, pneumonia	RT-PCR, confirmed with hybridization	NR	NR	NR	NR	NR	[160]
	2009	<2	460	13	NR	NR	NR	Real-time RT-PCR	NR	NR	NR	NR	0	[161]
	2010–2013	<76	735	42	59.5	40	Throat (pharyngitis),nasopharynx (nasopharyngitis), sinuses (sinusitis), larynx (laryngitis) and trachea (tracheitis)	Multiplex PCR	NR	NR	NR	NR	NR	[162]
	2010–2014	<80	351	46	NR	NR	NR	RT-PCR	NR	NR	NR	NR	11 (23.9%)	[115]
	2016	0-60	305	77	42.9	57.1	NR	RT-PCR	52 (67.5%)	NA1, ON1	25 (32.5%)	Twelve (55%) strains clustered with the BA10 and the rest (45%) were clustered into three groups of untyped strains that do not belong to any of the known group B genotypes	17 (22%)	[67]
Jordan	2002–2004	<2	200	25	72	28	Bronchiolitis, hypoxemia, retractions, tachypnea	Immunofluorescence analysis	NR	NR	NR	NR	NR	[3]
	2003–2004	<5	326	140	NR	NR	NR	RT-PCR	94 (70%)	NP2, NP4	41 (30%)	NP1, NP3	67 (48%)	[76]
	2007	<5	728	467	55	45	Cough, poor appetite, trouble breathing, post-tussive emesis, and wheezing	RT-PCR	NR	NR	NR	NR	126 (27%)	[163]
	2010–2013	<2	3168	1394	60	40	ALRTI symptoms and fever	RT-PCR	NR	NR	NR	NR	669 (48%)	[10]
Tunisia	2000–2002	<5	815	176	NR	NR	NR	RT-PCR	7 (17.5%)	NR	33 (82.5%)	NR	NR	[78]
	2000–2002	<35 days	268	62	58.7	41.3	Cough, wheezing and dyspnoea, and cyanosis and apnoea	Direct immunofluorescence assay and RT PCR	2 (13%)	NR	13 (87%)	NR	NR	[79]
	2005	<1	81	81	56.8	43.2	NR	RT-PCR	9 (11.1%)	NR	60 (74.1%)	NR	NR	[80]
	2009–2010		368	157	NR	NR	NR	Indirect immunofluorescence assay and PCR	NR	NR	NR	NR	NR	[163]
	2013–2014	<5	372	123	NR	NR	ALRTI symptoms by wheezing, tachypnea, and signs of respiratory distress such as nasal flaring, intercostal/subcostal retractions, and central cyanosis.	multiplex RT-PCR	NR	NR	NR	NR	NR	[164]
	2013–2014	<1	515	171	NR	NR	NR	multiplex qRT-PCR	NR	NR	NR	NR	73 (42.7%)	[165]
	2003–2015	<=5	5131	1769	NR	NR	ALRTI symptoms including wheezing, tachypnea, and signs of respiratory distress such as nasal flaring, intercostal/subcostal retractions, and central cyanosis	Direct immunofluorescence assay	NR	NR	NR	NR	NR	[166]
Algeria	2010–2011	<2	117	56	NR	NR	Fever or hypothermia associated with at least one of the following symptoms: polypnoea, wheezing, abnormalities in pulmonary listening or friction	RT-PCR	10 (17.9%)	NR	46 (82.1%)	NR	19 (33.9%)	[81]
Morocco	2010–2011	2-59 months	683	124	62.9	37.1	Breathing difficulty, chest indrawing,	Real-time PCR	NR	NR	NR	NR	54 (43.5%)	[167]
	2010–2011	<5	700	126	NR	NR	Cough, breathing difficulty and increased respiratory rate (RR) according to age and chest indrawing.	RT-PCR	NR	NR	NR	NR	NR	[168]
Pakistan	2009–2012	<5	223	223	NR	NR	Fever, cough, wheezing, poor appetite, shortness of breath, sore throat	Monoplex RT-PCR assay	NR	NR	NR	NR	NR	[169]
	2010–2011	<5	797	236	54.2	45.8	cough, fever and sore throat	RT-PCR	206 (87.3%)	NA1, GA2	30 (12.7%)	BA-9, BA-10 and the new BA-13 genotype	NR	[85]
	2011–2012	<5	610	119					91 (76.5%)		28 (23.5%)		NR	
	2012–2013	<5	534	117					70 (59.8%)		47 (40.2%)		NR	
	2010–2011	6 weeks to 2 years	169	30	66.7	33.3	Severe pneumonia	PCR	NR	NR	NR	NR	NR	[170]
	2011–2012	<2	155	104	NR	NR	coughing, runny nose, plus one of the following: wheezing, tachypnea, dyspnea, cyanosis, intercostal retractions, congestion, and/or crepitations on lung auscultation	NR	59 (58.9%)	NR	42 (41.1%)	NR	7 (6.7%)	[171]
	2011–2012	<2	105	75	53.3	46.7	Bronchiolitis or pneumonia	Nested RT-PCR	71 (94.7%)	GA2, NA1	4 (5.3%)	BA	4 (5.3%)	[172]
	2012–2013	NR	130	23	NR	NR	NR	NR	NR	NR	NR	NR	NR	[173]
	2014–2015	NR	712	48	NR	NR	NR	Magpix platform	NR	NR	NR	NR	NR	[174]
Total Number			69,981	17,106					2643		1561		1336	

NR: Not reported.
